# Joint Effects of Lifestyle Habits and Heavy Metals Exposure on Chronic Stress Among U.S. Adults: Insights from NHANES 2017–2018

**DOI:** 10.3390/jox15010007

**Published:** 2025-01-07

**Authors:** Esther Ogundipe, Emmanuel Obeng-Gyasi

**Affiliations:** 1 Department of Built Environment, North Carolina A&T State University, Greensboro, NC 27411, USA; 2 Environmental Health and Disease Laboratory, North Carolina A&T State University, Greensboro, NC 27411, USA

**Keywords:** allostatic load (AL), environmental pollutants, alcohol consumption, Bayesian kernel machine regression (BKMR), cumulative physiological stress

## Abstract

Background: Chronic stress, characterized by sustained activation of physiological stress response systems, is a key risk factor for numerous health conditions. Allostatic load (AL), a biomarker of cumulative physiological stress, offers a quantitative measure of this burden. Lifestyle habits such as alcohol consumption and smoking, alongside environmental exposures to toxic metals like lead, cadmium, and mercury, were individually implicated in increasing AL. However, the combined impact of these lifestyle habits and environmental factors remains underexplored, particularly in populations facing co-occurring exposures. This study aims to investigate the joint effects of lifestyle habits and environmental factors on AL, using data from the NHANES 2017–2018 cycle. By employing linear regression and Bayesian Kernel Machine Regression (BKMR), we identify key predictors and explore interaction effects, providing new insights into how cumulative exposures contribute to chronic stress. Results from BKMR analysis underscore the importance of addressing combined exposures, particularly the synergistic effects of cadmium and alcohol consumption, in managing physiological stress. Methods: Descriptive statistics were calculated to summarize the dataset, and multivariate linear regression was performed to assess associations between exposures and AL. BKMR was employed to estimate exposure–response functions and posterior inclusion probabilities (PIPs), focusing on identifying key predictors of AL. Results: Descriptive analysis indicated that the mean levels of lead, cadmium, and mercury were 1.23 µg/dL, 0.49 µg/dL, and 1.37 µg/L, respectively. The mean allostatic load was 3.57. Linear regression indicated that alcohol consumption was significantly associated with increased AL (β = 0.0933; 95% CI [0.0369, 0.1497]; *p* = 0.001). Other exposures, including lead (β = −0.1056; 95% CI [−0.2518 to 0.0408]; *p* = 0.157), cadmium (β = −0.0001, 95% CI [−0.2037 to 0.2036], *p* = 0.999), mercury (β = −0.0149; 95% CI [−0.1175 to 0.0877]; *p* = 0.773), and smoking (β = 0.0129; 95% CI [−0.0086 to 0.0345]; *p* = 0.508), were not significant. BKMR analysis confirmed alcohol’s strong importance for AL, with a PIP of 0.9996, and highlighted a non-linear effect of cadmium (PIP = 0.7526). The interaction between alcohol and cadmium showed a stronger effect on AL at higher exposure levels. In contrast, lead, mercury, and smoking demonstrated minimal effects on AL. Conclusions: Alcohol consumption and cadmium exposure were identified as key contributors to increased allostatic load, while other exposures showed no significant associations. These findings emphasize the importance of addressing lifestyle habits and environmental factors in managing physiological stress.

## 1. Introduction

Chronic stress, unlike acute stress, results from sustained exposure to stressors over time, leading to profound physiological dysregulation [1]. Prolonged activation of stress response systems, particularly the hypothalamic–pituitary–adrenal (HPA) axis and the sympathetic nervous system, is linked to a range of adverse health outcomes, including cardiovascular disease (CVD), metabolic disorders, and psychiatric conditions [2,3,4]. The concept of allostatic load, as introduced by McEwen and Stellar, provides a framework to quantify the cumulative physiological burden of chronic stress through biomarkers such as cortisol, blood pressure, and inflammatory markers [5]. An elevated allostatic load is indicative of increased susceptibility to chronic diseases and accelerated biological aging.

Lifestyle habits, such as excessive alcohol consumption (wine, beer, spirits) and cigarette smoking, are well-established contributors to increased allostatic load [6]. Chronic alcohol intake disrupts HPA axis function, leading to dysregulated cortisol production and heightened physiological strain on metabolic and cardiovascular systems [7,8]. Similarly, cigarette smoking induces chronic activation of the sympathetic nervous system, elevating heart rate and blood pressure while promoting systemic inflammation and oxidative stress, which collectively exacerbate allostatic load [9,10].

Heavy metal exposure is a critical environmental factor that exacerbates chronic stress. These metals, prevalent in industrial and environmental settings, were shown to interfere with neuroendocrine, immune, and cardiovascular systems thereby contributing to chronic stress responses [11]. Lead exposure was linked to impaired cortisol regulation, cognitive dysfunction, and hypertension, while cadmium induces oxidative stress and inflammation, increasing the physiological burden on the body [12]. Mercury, though less studied, interferes with neuroendocrine pathways and may amplify stress by promoting systemic inflammation and oxidative damage [13,14].

The interplay between environmental factors and lifestyle habits is particularly concerning, as they can have a synergistic impact on allostatic load, amplifying the physiological toll of chronic stress [15]. Individuals exposed to environmental toxins such as lead, cadmium, or mercury who also engage in high-risk lifestyle habits like smoking or excessive alcohol use may experience an intensified disruption of the HPA axis, higher oxidative stress, and more severe inflammation compared to those with exposure to only one of these stressors [16]. Combined exposures not only increase stress biomarkers, such as cortisol and blood pressure, but also accelerate the progression of chronic diseases by overwhelming the body’s ability to maintain homeostasis [5]. Notably, individuals from socioeconomically disadvantaged backgrounds are disproportionately exposed to both lifestyle stressors and environmental exposures, amplifying their risk of chronic stress and associated health outcomes [17].

Although substantial research has examined the independent effects of lifestyle habits and environmental factors on stress physiology, the interaction between these exposures remains underexplored. Given that both lifestyle habits (e.g., smoking and alcohol use) and environmental toxicants (e.g., metals) often co-occur, particularly in vulnerable populations, their combined effects may synergistically exacerbate allostatic load [15].This study aims to investigate the joint impact of alcohol consumption, smoking, and toxic metal exposure on chronic stress, as measured by allostatic load, to elucidate the complex interactions between lifestyle habits and environmental risk factors for stress-related diseases. We applied Bayesian Kernel Machine Regression (BKMR) to examine complex, non-linear interactions among exposures. By emphasizing the cumulative impact of metals and behavioral factors, this research provides a more comprehensive understanding of stress-related risk factors and highlights opportunities for targeted public health interventions.

## 2. Materials and Methods

### 2.1. Study Design

This study utilized data from the National Health and Nutrition Examination Survey (NHANES) for the years 2017–2018. NHANES is a cross-sectional survey designed to assess the health and nutritional status of a nationally representative sample of the non-institutionalized U.S. population. Conducted by the U.S. Centers for Disease Control and Prevention (CDC), the survey employs a multi-stage, stratified sampling design, and data are collected in two-year cycles. The dataset includes participants from all 50 states and the District of Columbia. Participants provided informed consent, underwent physical examinations, and participated in interviews. Blood samples were collected and sent to laboratories for analysis. Demographic information such as age, sex, and ethnicity were obtained through a Computer-Assisted Personal Interview (CAPI) system, ensuring accurate data collection. The survey protocols were approved by the Institutional Review Board at the National Center for Health Statistics (NCHS), CDC.

### 2.2. Variables and Covariates

The primary outcome variable for this study was allostatic load, a measure of chronic stress. Predictor variables included lifestyle habits—such as smoking and alcohol consumption—and environmental exposures to heavy metals (lead, cadmium, and mercury). Covariates adjusted in the model included demographic factors such as age, sex, ethnicity, and income.

### 2.3. Inclusion and Exclusion Criteria

Participants aged 18–80 years with complete data on heavy metals exposure, smoking/alcohol consumption data, and AL biomarkers were included. Exclusion criteria encompassed individuals with incomplete data or pre-existing conditions significantly influencing stress biomarkers.

### 2.4. Measurement of Heavy Metals (Lead, Cadmium, and Mercury)

The levels of heavy metals in whole blood samples were measured using inductively coupled plasma dynamic reaction cell-mass spectrometry (ICP-DRC-MS). These assays were conducted by the CDC’s Division of Laboratory Sciences at the National Center for Environmental Health. Detailed laboratory procedures regarding quality control and quality assurance can be found on the NHANES website [18].

The concentrations of lead, cadmium, and mercury in blood were measured using inductively coupled plasma mass spectrometry (ICP-MS), a highly sensitive method that quantifies metal content with precision. Blood samples were prepared by diluting 1-part whole blood with 1-part water and 48-parts diluent containing tetramethylammonium hydroxide (TMAH) and Triton X-100™. This preparation facilitated the release of metals bound to red blood cells, reduced ionization suppression by the biological matrix, and prevented clogging of the system. Internal standards (rhodium, iridium, and tellurium) were used to account for instrument drift and matrix variability.

The limits of detection (LOD) for the metals analyzed in this study were as follows: 0.07 µg/dL for lead, 0.10 µg/L for cadmium, and 0.28 µg/L for total mercury. Samples with values below the LOD were assigned an imputed value equal to the LOD divided by the square root of 2 (LOD/√2) to minimize bias.

#### Absorption, Distribution, Metabolism, and Excretion (ADME)

Lead is primarily absorbed through inhalation and ingestion, with absorption rates varying based on age and nutritional status. Children absorb lead more efficiently than adults, making them particularly vulnerable. Once absorbed, lead circulates in blood, binds to red blood cells, and is distributed to soft tissues and bones. The majority of lead is stored in bones, where it can remain for decades, acting as a reservoir that slowly releases lead back into the bloodstream. Excretion of lead occurs primarily through urine and feces, but the process is slow, leading to its long biological half-life.

Cadmium exposure occurs through inhalation of cigarette smoke and ingestion of contaminated food. Smokers have blood cadmium levels approximately twice as high as non-smokers due to direct inhalation. Cadmium is absorbed efficiently in the lungs but less so in the gastrointestinal tract. Once absorbed, cadmium is transported to the liver, where it binds to metallothionein and is distributed primarily to the kidneys, where it accumulates over time. Excretion is minimal and occurs via urine, making cadmium a persistent toxin with a long biological half-life.

Mercury exposure is largely dietary, primarily from the consumption of fish contaminated with methylmercury. Methylmercury is highly absorbed in the gastrointestinal tract and distributed throughout the body, including to the brain and central nervous system, where it can cross the blood–brain barrier. It is metabolized into inorganic mercury, which is excreted slowly through urine and feces. The half-life of mercury varies depending on its form, with methylmercury having a half-life of approximately 50 days in the human body.

### 2.5. Alcohol and Smoking Assessment

Alcohol consumption was assessed based on the NHANES Alcohol Use Questionnaire, which includes data on both lifetime and current alcohol use. Smoking behavior on the other hand was evaluated using data on cigarette use, with the focus on current smoking status [19]. The frequency of alcohol consumption was assessed using the self-reported questionnaire that inquired about participants’ alcohol use over the past year. The questionnaire included 11 categories, ranging from “Every day” to “Never in the last year”, providing a detailed stratification of drinking behaviors. These categories captured a wide spectrum of consumption patterns, including daily drinkers, those who consumed alcohol nearly every day, several times a week, weekly, monthly, or only a few times in the past year. The responses also included a category for participants who reported no alcohol consumption in the past year. This detailed stratification enabled a nuanced analysis of alcohol consumption frequency and its relationship with other variables in the study.

Smoking behavior was assessed based on the average number of cigarettes smoked per day during the past 30 days, as reported by participants in the Smoking—Cigarette Use questionnaire. Responses ranged from 0 to 60 cigarettes per day, capturing a wide spectrum of smoking intensity, from non-smokers to heavy smokers. This measure provided a detailed characterization of smoking behavior, enabling the analysis of smoking frequency and its potential association with other variables in the study.

### 2.6. Statistical Analysis

#### 2.6.1. Descriptive Statistics and Regression Analysis

Descriptive statistics were used to summarize key demographic variables, including age, sex, and ethnicity. Linear regression models were applied to evaluate the relationship between the outcome (allostatic load) and predictor variables (alcohol/smoking consumption and environmental exposures), while adjusting for covariates. Complete data were used for the analysis.

#### 2.6.2. Bayesian Kernel Machine Regression (BKMR)

To assess the combined impact of multiple contaminants, Bayesian Kernel Machine Regression (BKMR) was employed. BKMR is a flexible, non-linear regression method that allows for the detection of interaction effects among exposures. Unlike traditional linear regression models, BKMR can model complex, non-linear relationships between contaminants and health outcomes. The BKMR equation is shown below as follows:

$$Y_{i}=h\left(z_{i}\right)+x_{i}^{T}\beta +\varepsilon_{i},$$

where $Y_{i}$ represents a health outcome, and $z_{i}={\left(z_{i1}\ldots ,\;z_{iM}\right)}^{T}$ is a vector M exposure variables. The term $x_{i}$ includes a set of potential confounding variables and $\varepsilon_{i}$ is an independent and identically distributed random error term assumed to follow a normal distribution, $\varepsilon_{i}\sim N(0,\;\sigma^{2})$.

Exposure–response functions were modeled non-parametrically using Gaussian kernels. Markov Chain Monte Carlo (MCMC) sampling was used to estimate the model parameters and exposure–response relationships, with a total of 5000 iterations to ensure convergence.

Statistical analysis was conducted using R software (version 4.2.3; R Foundation for Statistical Computing, Vienna, Austria), and a significance level of 0.05 was used for non-Bayesian analyses.

## 3. Results

### 3.1. Descriptive Statistics

The descriptive statistics for the continuous variables, including lead, cadmium, mercury, allostatic load (AL), and age, are presented in Table 1. The geometric mean levels of lead and mercury were 1.05 µg/dL and 1.42 µg/L, respectively, while the geometric mean level of cadmium was 0.370 µg/dL. The allostatic load, which represents the cumulative physiological burden, had a geometric mean value of 3.43, with a standard deviation of 1.16, based on 1995 observations. The geometric mean age of participants in the sample was 43.1 years, ranging from 18 to 80 years.

The categorical variables, including sex and race/ethnicity, are summarized in Table 2. The sample was almost evenly split by sex, with 50.98% male and 49.02% female participants. In terms of race/ethnicity, 37.22% of the sample identified as Non-Hispanic White, followed by 23.45% Non-Hispanic Black, and 13.79% Mexican American.

### 3.2. Linear Regression Results

A multivariate linear regression analysis was conducted to assess the association between alcohol consumption, smoking, lead, cadmium, and mercury levels with allostatic load (AL), adjusting for age, gender, race/ethnicity, and income. The results of this analysis are presented in Table 3.

Alcohol consumption was found to have a statistically significant positive association with allostatic load (β = 0.0933, *p* = 0.001), indicating that higher alcohol consumption is associated with higher levels of physiological stress. Smoking, although positively associated with allostatic load (β = 0.0129), was not statistically significant (*p* = 0.508).

Among the environmental toxicants, none demonstrated a statistically significant relationship with allostatic load. Lead showed a negative but non-significant association (β = −0.1056, *p* = 0.157), and cadmium and mercury also had no significant effects (β = −0.0001, *p* = 0.999; β = −0.0149, *p* = 0.773, respectively).

### 3.3. Assessing Relationships Among Key Variables: Spearman Correlation Matrix Analysis

**Figure 1:**
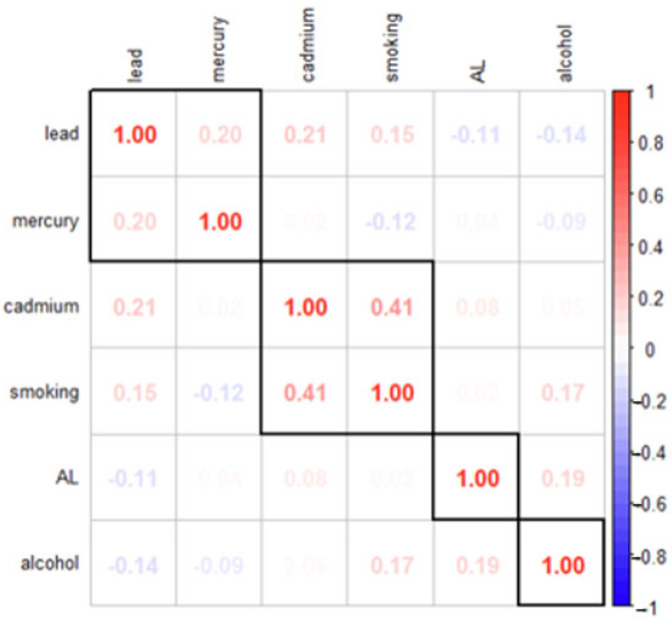
Spearman correlation matrix depicting relationships between variables of interest.

To evaluate the relationships between the continuous variables in the dataset, a Spearman correlation matrix was constructed (Figure 1). The Spearman correlation coefficient, a non-parametric measure of rank correlation, assesses the strength and direction of monotonic relationships between variables. This method is particularly suitable when the data do not meet the assumptions of normality required for Pearson’s correlation.

The variables examined in this analysis include lead, mercury, cadmium, smoking, allostatic load (AL), and alcohol consumption. Positive correlations indicate that as one variable increases, the other tends to increase as well, while negative correlations suggest an inverse relationship. The strength of the correlation is indicated by the absolute value of the coefficient, where values close to 1 indicate a strong relationship and values near 0 indicate little to no relationship.

This matrix illustrates the pairwise Spearman correlation coefficients between the variables, representing the strength and direction of monotonic relationships. Positive correlations are shown in red, while negative correlations are shown in blue, with the intensity reflecting the magnitude of the correlation.

### 3.4. Evaluating Variable Importance in BKMR: Insights from Posterior Inclusion Probabilities (PIPs)

In Bayesian Kernel Machine Regression (BKMR), the Posterior Inclusion Probability (PIP) quantifies the likelihood that each variable contributes to the model, providing a measure of variable importance. This analysis divides variables into groups based on related exposure or lifestyle habits, and both group PIP and conditional PIP values are used to assess the influence of each variable within the group. The group PIP represents the overall importance of a group of variables, while the conditional PIP (condPIP) evaluates the importance of individual variables within the group, conditional on their inclusion.

In the analysis presented in Table 4, the PIP results are divided into two groups.

- Group 1: Includes environmental pollutants (lead, cadmium, and mercury).
- Group 2: Includes lifestyle habits (alcohol consumption and smoking).
- Group 1 (environmental pollutants) has a moderate group PIP of 0.3864, indicating a moderate likelihood that these pollutants influence allostatic load. The pollutants are as follows:
  o Cadmium shows the highest conditional PIP (0.7526), suggesting that cadmium is the most important pollutant in predicting allostatic load in this model.
  o Lead has a lower conditional PIP (0.1749), suggesting less importance compared to cadmium.
  o Mercury has the lowest conditional PIP (0.0725), indicating minimal contribution to the model.
- Group 2 (lifestyle habits) shows a high group PIP of 0.9988, suggesting that lifestyle habits play a significant role in predicting allostatic load.
  o Alcohol consumption exhibits a very high conditional PIP (0.9996), indicating near-certain importance in the model.
  o Smoking, by contrast, has a very low conditional PIP (0.0004), suggesting that smoking plays a negligible role compared to alcohol consumption in this model.

These results emphasize the strong influence of alcohol consumption and cadmium exposure on allostatic load, while other variables such as lead, mercury, and smoking contribute less to the overall model. The group PIP and conditional PIP values provide insights into the relative importance of different exposures and lifestyle habits in relation to physiological stress.

### 3.5. Exposure–Response Analysis: Univariate Associations with Allostatic Load Using BKMR

**Figure 2:**
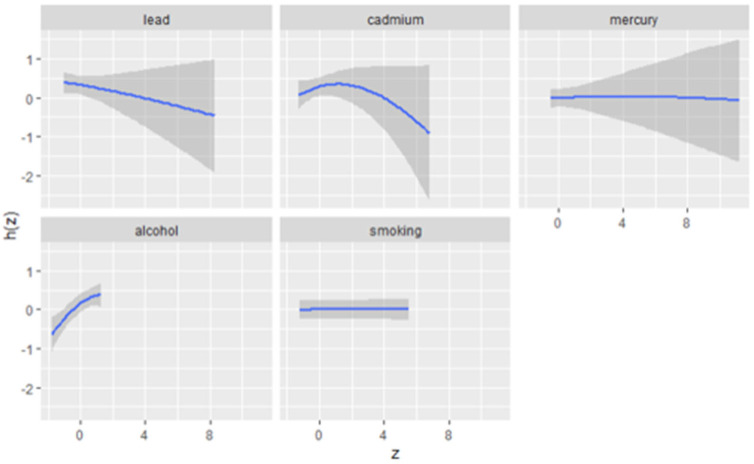
Univariate exposure–response functions (blue line) and 95% credible interval (grey area) for association between single metal/lifestyle factor when other metals and lifestyle factor exposures are fixed at median. Adjusted for age, gender, race/ethnicity, and income.

Figure 2 presents the univariate exposure–response functions, estimated using BKMR, for the association between each individual exposure (lead, cadmium, mercury, alcohol, and smoking) and allostatic load. These exposure–response curves depict the relationship between each exposure and the health outcome while holding all other exposures fixed at their median values. The shaded areas represent the 95% credible intervals, providing an estimate of the uncertainty around the exposure–response relationship.

The results show distinct patterns for the various exposures.

- Lead: The exposure–response curve suggests a negative association between lead exposure and allostatic load. As lead levels increase, allostatic load decreases, although the association is relatively weak, as indicated by the wide credible interval at higher exposure levels.
- Cadmium: The relationship between cadmium exposure and allostatic load appears non-linear. The curve initially rises, suggesting a positive association with moderate cadmium exposure, before leveling off and slightly decreasing at higher exposure levels. The relatively narrow credible interval indicates greater certainty in this relationship.
- Mercury: There is no clear association between mercury exposure and allostatic load, as the curve remains flat across exposure levels. The wide credible interval, especially at higher exposure levels, indicates substantial uncertainty in the estimate.
- Alcohol Consumption: The exposure–response function shows a clear positive association between alcohol consumption and allostatic load. As alcohol consumption increases, allostatic load rises, with a narrow credible interval suggesting strong evidence for this relationship.
- Smoking: The response curve for smoking is nearly flat, indicating no significant relationship between smoking and allostatic load when other exposures are held constant. The narrow credible interval suggests little uncertainty around this null association.

### 3.6. Exploring Joint and Quantile-Based Exposure–Response Functions in BKMR: Dual Influences on Allostatic Load

**Figure 3:**
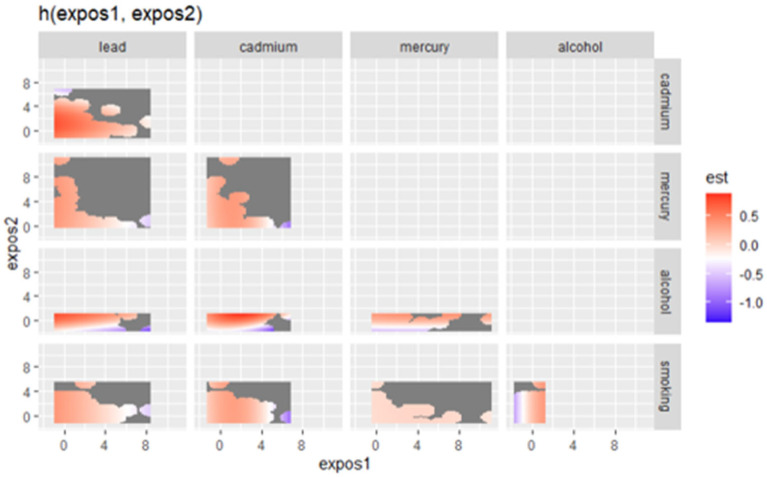
Bivariate exposure–response function of metals with AL. Adjusted for age, gender, race/ethnicity, and income.

Figure 3 presents the bivariate exposure–response functions estimated from Bayesian Kernel Machine Regression (BKMR), illustrating the joint effects of two exposures (lead, cadmium, mercury, alcohol, and smoking) on allostatic load (AL). The figure depicts how changes in combinations of two exposures simultaneously influence allostatic load, while the other exposures are held at their median values. The color scale represents the magnitude of the estimated response, where red areas indicate positive associations with AL and blue areas represent negative associations. Gray areas reflect regions where there is little evidence of an effect or higher uncertainty.

#### Quantile-Based Bivariate Exposure–Response Functions

**Figure 4:**
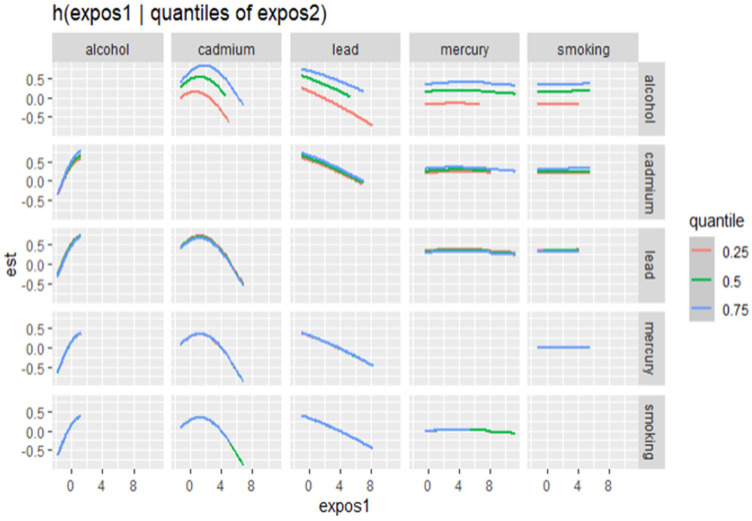
Bivariate exposure–response function of metals with AL: investigating exposure–response function with varying quantiles of second exposure, while other exposures are fixed. Orange, green, and blue are the 0.25, 0.5, and 0.75 quantile of the second exposure. Analysis adjusted for age, gender, race/ethnicity, and income.

Figure 4 presents the quantile-based bivariate exposure–response functions from the Bayesian Kernel Machine Regression (BKMR) analysis. In this figure, the association between one exposure (shown on the x-axis) and allostatic load (AL) is explored at different quantiles (0.25, 0.50, and 0.75) of a second exposure, while holding all other exposures constant at their median values. This method allows us to observe how the relationship between an individual exposure and allostatic load changes as the second exposure varies across its distribution.

### 3.7. Overall Exposure Effect of Multiple Pollutants on Allostatic Load Across Quantiles

**Figure 5:**
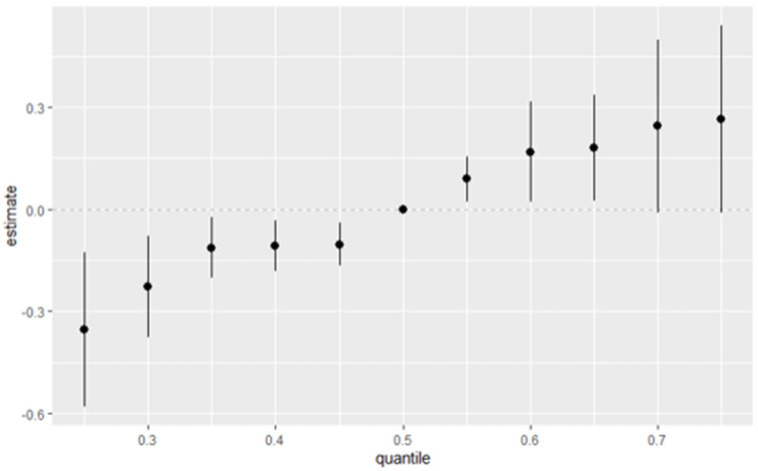
The overall effect of combined lifestyle factors and metals and the estimated change in chronic stress (with estimates and 95% credible intervals) at specific percentiles relative to their 50th percentile level. Adjusted for age, gender, race/ethnicity, and income.

Figure 5 summarizes the overall health effects of multiple exposures (multi mixtures) on allostatic load (AL) across various percentiles of exposure. The figure captures the estimated effect of combined exposures at different percentiles, ranging from the 25th percentile (0.25) to the 75th percentile (0.75). Each point represents the estimated effect size at a given quantile, with vertical lines indicating the 95% credible intervals, which reflect the uncertainty around the estimates.

### 3.8. Single-Exposure Effects on Allostatic Load Across Exposure Quantiles: Metals and Lifestyle Habits

**Figure 6:**
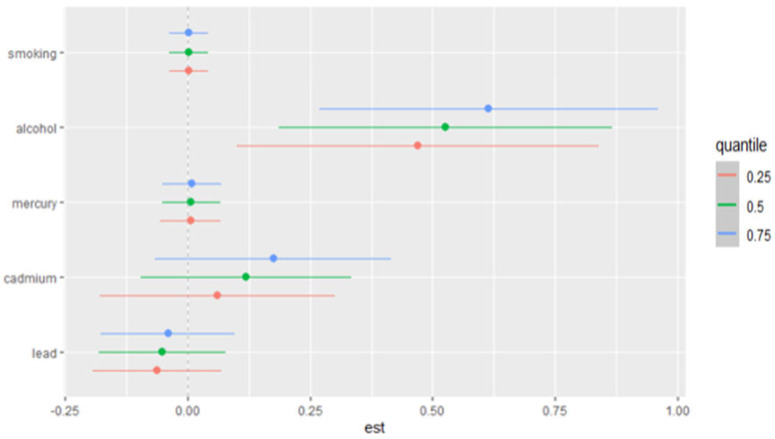
Single-exposure AL effects (95% CI), defined as the change in the response associated with a change in a particular exposure from its 25th to its 75th quantile, where all of the other exposures are fixed at a specific quantile (0.25, 0.50, or 0.75). Adjusted for age, gender, race/ethnicity, and income.

Figure 6 presents the single-variable effects of metals and lifestyle habits (smoking and alcohol consumption) on allostatic load (AL) at increasing quantiles of exposure. The estimates of the effect size (est) are displayed along the x-axis, with horizontal lines representing the 95% credible intervals. Different colors represent the effects at various quartiles (e.g., 25th, 50th, 75th percentiles), allowing for a comparison of how the relationship between each exposure and allostatic load changes across the distribution of exposure.

### 3.9. Interaction Effects of Metals and Lifestyle Habits on Allostatic Load

**Figure 7:**
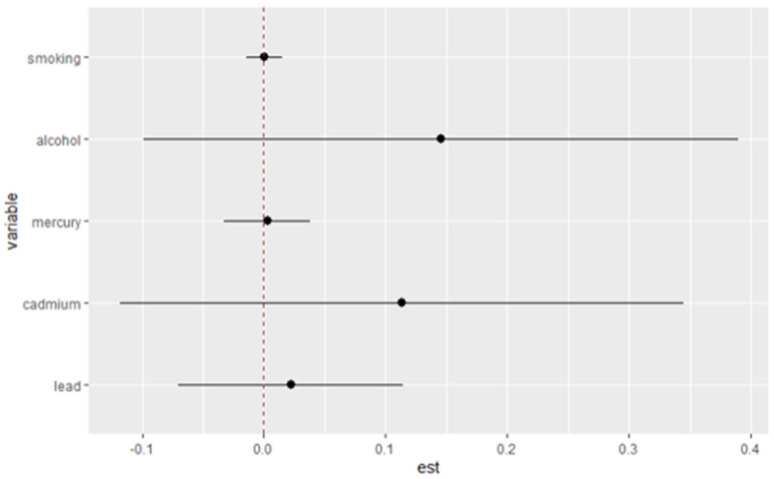
Single-variable interaction terms (black dots and lines represent estimates and 95 % credible intervals) for mercury, cadmium, lead, alcohol consumption, and smoking. Adjusted for age, gender, race/ethnicity, and income.

Figure 7 illustrates the single-variable interaction terms for metals (mercury, cadmium, and lead) and lifestyle habits (alcohol consumption and smoking) in their relationship with allostatic load (AL).

## 4. Discussion

Our study investigated the joint effects of lifestyle habits (alcohol, smoking) and environmental exposures (lead, cadmium, mercury) on allostatic load (AL), a marker of chronic stress. The descriptive statistics provided an overview of the study population and key variables. Among the environmental exposures, lead, cadmium, and mercury levels showed variability, with mean concentrations of 1.23 µg/dL, 0.49 µg/dL, and 1.37 µg/L, respectively. Mercury exhibited the highest variability, as indicated by its standard deviation (2.41 µg/L) and maximum value of 63.64 µg/L. Allostatic load (AL), a measure of cumulative stress, had a mean of 3.57 with a range from 0 to 10, reflecting variability in chronic stress levels among participants. The mean age of participants was approximately 50 years, with a range from 18 to 80 years, indicating a diverse age distribution.

Regarding demographic characteristics, the sample was nearly evenly split by sex, with 50.98% male and 49.02% female participants. In terms of race/ethnicity, Non-Hispanic Whites constituted the largest group (37.22%), followed by Non-Hispanic Blacks (23.45%) and Mexican Americans (13.79%). Other Hispanic participants (9.23%), Non-Hispanic Asians (10.81%), and those identifying as Other Race/Multi-Racial (5.51%) made up the remainder of the sample. This distribution highlighted the diversity of the study population, which was essential for understanding the interplay between environmental exposures, lifestyle habits, and health outcomes across demographic groups.

The Spearman correlation plot provided insights into the monotonic relationships between variables, capturing the strength and direction of associations regardless of whether the relationships are linear. This method is particularly useful for understanding rank-based relationships, as it is robust against outliers and non-normal data distributions. Positive correlations were observed among the heavy metals, including lead, mercury, and cadmium. For instance, lead and cadmium showed a moderate positive correlation (r = 0.21), while weaker correlations were observed between lead and mercury (r = 0.20) and between mercury and cadmium (r = 0.21). These findings suggested that individuals exposed to one heavy metal were likely exposed to others, potentially due to shared environmental sources or co-occurring risk factors.

Lifestyle habits, such as smoking and alcohol consumption, also demonstrated some associations with the other variables. Smoking was positively correlated with cadmium (r = 0.41) and lead (r = 0.20), reflecting known links between smoking and increased heavy metal exposure, particularly cadmium from cigarette smoke. In contrast, alcohol consumption showed weak or negligible correlations with other variables, including heavy metals and allostatic load (AL), indicating that alcohol might not co-occur strongly with these exposures in the dataset. Allostatic load exhibited a weak negative correlation with lead (r = −0.11) and negligible correlations with mercury and cadmium. It was weakly positively correlated with alcohol (r = 0.19) and smoking (r = 0.08), indicating minor associations between these lifestyle habits and stress-related health impacts.

While generally weak, the observed correlations suggested modest monotonic relationships among heavy metals, lifestyle habits, and allostatic load. Positive correlations, such as those between smoking and cadmium, indicated that as the rank of one variable increased, the rank of the other also increased, whereas negative correlations, such as between lead and allostatic load, suggested an inverse relationship. Since Spearman correlation measured monotonicity, the coefficients reflected ordered relationships but did not necessarily imply linearity. These findings highlighted modest monotonic relationships among the variables, with smoking appearing to be a key lifestyle factor linked to cadmium and lead exposure, while alcohol showed weaker associations. The correlations between heavy metals and allostatic load were weak, suggesting that their contributions to stress-related health outcomes were likely complex and influenced by other interacting factors but a higher level analysis such as BKMR was needed to confirm this.

Both linear regression and BKMR analyses identified alcohol and cadmium as the primary contributors to increased AL. Alcohol showed a strong positive association (β = 0.0933; *p* = 0.001) in linear regression, with a PIP of 0.9996 in BKMR, demonstrating its importance in explaining AL. Cadmium, on the other hand, exhibited a non-linear effect in the BKMR analysis and was the second most important factor in explaining AL (PIP = 0.7526). Lead, mercury, and smoking had minimal impact, with low PIP values. Combined exposures significantly elevated AL, with alcohol being the dominant factor.

The interactions between variables (cadmium–alcohol) highlight the complexity of cumulative exposures. There were also complex relationships between lifestyle habits and environmental exposures. These insights underscore the importance of addressing both lifestyle and environmental risk factors simultaneously in public health strategies [20].

Comparison with previous studies confirms the role of lifestyle and environmental factors in stress, but our study adds depth by highlighting alcohol’s predominant influence and cadmium’s non-linear relationship with AL. This underscores the importance of assessing combined exposures rather than individual factors, particularly in understanding the stress-related effects of alcohol, which aligns with its known disruption of the HPA axis and cortisol elevation [7].

Previous studies have found that alcohol exacerbates physiological stress, leading to increased blood pressure, heart rate, and inflammatory markers [21]. Our findings confirmed this strong association, with a nearly perfect inclusion probability (PIP = 0.9996) in the BKMR model. This is in line with previous research, although some studies have noted that moderate alcohol consumption may help reduce cardiovascular risk [22,23]. Our results emphasize that increased alcohol consumption impairs stress control, underscoring the negative effects of heavy drinking on AL [2]. This also highlights the need for targeted interventions, a point that is reflected in other studies focusing on reducing alcohol consumption to mitigate chronic disease risks [22].

Cadmium was the second most significant predictor of AL highlighted in our BKMR analysis (PIP = 0.7526) and demonstrated a complex non-linear relationship. This differs from other prior studies, which mostly reported linear relationships between cadmium exposure and health problems like cardiovascular disease [24]. That said, these studies examined one contaminant at a time, while ours explored cadmium relationships in a mixture. The non-linear response in our study, where moderate exposure levels had a stronger impact but plateaued at higher levels, may indicate adaptive physiological responses. This finding expands on previous research by suggesting that the body may deploy compensatory mechanisms at high exposure thresholds [24]. However, previous studies have consistently shown that cadmium exposure increases oxidative stress and inflammation, both of which elevate AL, a trend supported by our findings [25,26]. Our results suggest the need for a more refined approach to understanding the limits at which cadmium exposure becomes most harmful, contributing to the body of knowledge on environmental stressors [27].

Unlike other studies that have revealed stronger links between lead and mercury exposure and negative health outcomes, including cardiovascular and neurodegenerative diseases, our study found no significant impacts of either metal on AL [28]. In contrast to the clear links with cadmium, the low predictive importance of lead and mercury in our analysis may reflect lower exposure levels in the study population or could indicate that the interaction between these metals and other stressors (like alcohol) dampens their individual effects [29]. This finding aligns with some previous research, which has noted that the effects of heavy metals can be context-dependent or require long-term exposure to manifest [11]. Future studies are needed to clarify these interactions and determine specific thresholds where lead and mercury significantly influence AL.

Previous studies have found considerable connections between smoking and stress; however, our study found minor impacts on AL [30]. This disparity could be explained by lower smoking intensity or shorter duration among our study participants, or by the overshadowing effect of more dominant factors such as alcohol intake. Some studies have also found that smokers may participate in stress-relieving behaviors or have coping strategies that mitigate the physiological effects of smoking, which could explain the reduced link [31]. This discovery necessitates additional investigation into the various impacts of smoking on stress, particularly in the presence of other concurrent exposures.

### 4.1. Overall Combined Effect

Our analysis found that the combined impact of alcohol consumption, smoking, and heavy metal exposure significantly increased AL, highlighting the cumulative burden of multiple stressors on physiological stress. BKMR analysis also showed a strong combined effect, especially when lifestyle habits were assessed with environmental exposures, with a high group PIP value (0.9988) for lifestyle habits. This supports cumulative stress models, which suggest that multiple stressors, like environmental exposure and bad lifestyle choices, overwhelm the body, leading to dysregulated stress responses, increased inflammation, and higher AL [32,33].

Comparative studies similarly show that co-exposure to environmental pollutants and unhealthy lifestyle choices amplifies AL, raising chronic disease risk [29]. These findings highlight the need for integrated public health interventions targeting both lifestyle changes (smoking cessation, alcohol reduction) and environmental policies to reduce heavy metal exposure [34]. Addressing these factors together could mitigate the heightened physiological stress seen in populations facing this “double burden” [35].

### 4.2. Single-Variable Effects and Interaction Terms

#### 4.2.1. Single-Variable Effects

Alcohol exhibited a consistent and strong positive association with AL across all exposure levels. This means that irrespective of whether alcohol consumption was low or high, its effect on increasing AL was evident and sustained [33]. This consistent relationship suggests that alcohol acts as a significant stressor across different levels of exposure, likely through mechanisms involving cortisol dysregulation, immune function impairment, and increased oxidative stress [36].

Cadmium also displayed a strong single-variable effect on AL. The chart showed that cadmium’s impact on AL increased as exposure levels increased, indicating a physiologically impactful dose–response relationship [24,29]. This relationship might be due to cadmium’s role in inducing oxidative stress and inflammation, processes that become more damaging as exposure increases [37].

Comparatively, lead, mercury, and smoking showed weaker individual effects on AL. These findings diverge from some studies that have reported more significant impacts of lead and mercury on stress-related outcomes [28]. However, it suggests that in the presence of stronger stressors like alcohol and cadmium, the individual contributions of lead, mercury, and smoking might be less noticeable or could be interacting in ways that diminish their apparent effect in our data sample [32].

When comparing these results to previous research, studies examining the synergistic effects of environmental and lifestyle habits on stress-related health outcomes have found similar patterns [38]. Research has demonstrated that alcohol can potentiate the harmful effects of heavy metal exposure by impairing detoxification pathways and exacerbating oxidative stress, leading to higher AL [21]. Likewise, cadmium was shown to interact with other pollutants, amplifying stress responses, particularly in populations already at risk due to unhealthy lifestyle choices or socioeconomic factors [34].

#### 4.2.2. Interaction Terms

##### Alcohol and Allostatic Load

Alcohol showed strong interactions in all study analyses. Alcohol’s strong positive association with AL, as observed in the bivariate results and the single-exposure analysis, can be attributed to several biological mechanisms, particularly its influence on the HPA axis, inflammation, and immune response. In a study conducted by Thayer et al., it was revealed that alcohol disrupts the normal functioning of the HPA axis, a key regulator of the body’s stress response. In the same study, chronic alcohol consumption was shown to alter cortisol levels, leading to prolonged stress activation. Cortisol, the body’s primary stress hormone, is regulated by the HPA axis and plays a pivotal role in maintaining homeostasis; prolonged alcohol consumption can dysregulate this system, resulting in elevated cortisol levels and, consequently, increased allostatic load [36].

Additionally, alcohol promotes systemic inflammation, which further increases physiological stress [21]. Studies have shown that excessive alcohol intake increases pro-inflammatory cytokines, such as TNF-α and IL-6, contributing to chronic inflammation and immune dysfunction [39]. The immune response is also compromised by alcohol, weakening the body’s defense mechanisms and making it more susceptible to infections and stress-related diseases [39,40]. This dual effect, that is, the HPA axis dysregulation and inflammation, explains why alcohol was such a strong predictor of AL in this study [41].

Moreover, alcohol interacts with other exposures, such as heavy metals, to exacerbate physiological stress [21]. Alcohol impairs detoxification processes in the liver, which may lead to the accumulation of toxicants like cadmium, thus intensifying the body’s stress burden [27]. This interaction between lifestyle choice (alcohol) and environmental (cadmium) stressors was evident in the interaction terms, where alcohol and cadmium together significantly increased AL.

##### Cadmium’s Non-Linear Effect

Cadmium demonstrated complex interactions in the bivariate analysis, and the presence of interaction in the single-exposure analysis was also apparent. When interacting with other factors, cadmium’s non-linear effect on AL can be explained by varying biological responses at different exposure levels. At lower exposure levels, the body might engage adaptive responses, attempting to mitigate the toxic effects of cadmium through mechanisms like increased production of metallothionein, proteins that bind cadmium and reduce its bioavailability [34]. These adaptive mechanisms could prevent cadmium from exerting significant effects on AL at lower doses, explaining the relatively flat association observed in the exposure–response curve at lower exposure levels [42].

However, at higher cadmium exposure levels, these adaptive responses may become overwhelmed, leading to more pronounced toxic effects. Cadmium is known to induce oxidative stress by increasing reactive oxygen species (ROS) and depleting antioxidant defenses, such as glutathione [27]. This oxidative stress can cause cellular damage, contribute to systemic inflammation, and disrupt immune function [27]. Additionally, cadmium interferes with hormonal regulation, particularly by disrupting the endocrine system, which can lead to further dysregulation of stress responses, including the HPA axis [34].

This pattern suggests that there may be threshold levels at which cadmium exposure transitions from being manageable by the body to becoming toxic and significantly increasing physiological stress, as reflected in higher AL values at moderate to high exposures [43]. This non-linear effect also highlights the importance of investigating the biological tipping points at which environmental exposures become particularly harmful.

##### Effects of Metals and Allostatic Load

The distributions of lead, cadmium, and mercury in the study population differed. Lead and mercury exhibited wide variability, while cadmium levels were more uniformly distributed. This heterogeneity may influence their relative contributions to AL.

Our findings highlighted the significant cumulative burden of combined metal exposures on AL. While metals such as lead, cadmium, and mercury were initially analyzed individually, the BKMR results underscored their combined impact. The group PIP for environmental pollutants was moderate (0.3864), indicating the importance of considering these metals as a mixture. This aligns with studies like Cory-Slechta et al. (2004), which reported that co-exposure to lead and cadmium amplifies oxidative stress and the dysregulation of the hypothalamic–pituitary–adrenal (HPA) axis, both key contributors to elevated AL [44]. The synergistic effects observed in our study support the hypothesis that metal combinations exacerbate physiological stress more than individual exposures.

##### Cumulative Burden of Multiple Exposures

The combined exposures from lifestyle habits (alcohol, smoking) and environmental factors (lead, cadmium, mercury) significantly increased AL, suggesting that cumulative stress from multiple sources plays a critical role in chronic physiological burden. According to the allostatic overload model, chronic exposure to multiple stressors can lead to a wear-and-tear effect on the body’s regulatory systems [33]. These systems, including the HPA axis, immune system, and cardiovascular system, are tasked with maintaining homeostasis in the face of ongoing stress [45]. When exposed to multiple stressors simultaneously, such as living in a polluted environment while engaging in risky lifestyle choices, the body’s regulatory capacity is overwhelmed, leading to chronic stress and allostatic overload [25]. This overload manifests as increased AL, which is associated with a higher risk of developing chronic health conditions, such as cardiovascular disease, metabolic syndrome, and diabetes [23]. Our results showed that alcohol and cadmium were dominant contributors to AL, with weaker effects observed for lead and mercury. This aligns with some studies but diverges from others. For example, prior research has consistently shown that cadmium induces oxidative stress and systemic inflammation, corroborating our findings of its significant contribution [46]. However, studies examining lead and mercury often report stronger effects than observed in our dataset [44]. Differences may stem from population-level variations, exposure levels, or the use of different biomarkers. Additionally, the cumulative impact of alcohol on cadmium’s effects, as seen in our study, is supported by the prior literature emphasizing alcohol’s role in impairing detoxification pathways and exacerbating oxidative damage from metals. A study conducted by Brzoska et al. [47] investigated the interaction between cadmium and ethanol exposure in rats. The study revealed that even short-term ethanol consumption increased cadmium retention and accumulation in the body, particularly in the liver and kidneys. They observed that ethanol administration enhanced cadmium-induced changes in the metabolism of important elements such as zinc and copper, likely due to the ability of both substances to induce metallothionein production. The study demonstrates that alcohol consumption can exacerbate the toxic effects of cadmium by increasing its retention in the body and disturbing the metabolism of essential bio elements [47]. Our study’s finding that alcohol and cadmium have the strongest individual effects, but that the combined exposures result in an even greater increase in AL, supports the concept that cumulative exposures are more harmful than single stressors.

Multiple studies have demonstrated that individuals living in high-pollution areas who engage in unhealthy lifestyle choices (heavy drinking or smoking) experience worse health outcomes than those exposed to only one type of stressor [25]. The combined effects of metal pollutants and lifestyle choices may create a synergistic effect, where the overall burden on the body is greater than the sum of the individual exposures, further stressing the need for an integrated approach to managing public health risks [25].

### 4.3. Public Health Implications

The findings from this study have important implications for public health interventions aimed at reducing allostatic load and related stress conditions. The significant role of alcohol consumption and cadmium exposure in increasing AL suggests that addressing these two factors could yield significant public health benefits [48,49]. Interventions should focus on reducing alcohol consumption and minimizing exposure to environmental contaminants, particularly heavy metals like cadmium [50].

Public health campaigns should aim to raise awareness about the health risks of heavy drinking and educate communities about the dangers of environmental pollution [50]. Programs targeting at risk populations, such as those living in industrialized or heavily polluted areas, should emphasize both lifestyle modifications (reducing alcohol intake and smoking cessation) and environmental safety measures, such as minimizing exposure to cadmium through dietary and occupational precautions [11,34].

Additionally, the cumulative burden of stress from environmental and lifestyle exposures underscores the need for comprehensive public health policies that address multiple risk factors simultaneously [49]. Policies should focus on reducing environmental pollution such as stricter regulations on industrial emissions and better management of hazardous materials while also promoting healthier lifestyle choices [49]. This is particularly important in vulnerable populations, such as low-income communities, who are more likely to be exposed to both high levels of environmental contaminants and engage in unhealthy lifestyle choices due to limited access to healthcare and preventive services [51].

### 4.4. Study Limitations

Our study offers significant information including the use of nationally representative NHANES data. Additionally, the application of Bayesian Kernel Machine Regression allowed us to examine complex, non-linear interactions among multiple exposures, providing insights into cumulative stress effects from both environmental and lifestyle habits; however, this study is not without limitations. The primary limitation that was observed in our study is its cross-sectional design, which limits the capacity to draw causal inferences. While relationships between exposures and AL were discovered, it is impossible to say whether these exposures directly caused the increase in AL or whether other unmeasured factors played a role. Longitudinal research is required to further determine the temporal link between these exposures and chronic stress consequences. Another important limitation observed is the unmeasured confounders that were not accounted for in the analysis. For example, genetic predispositions, psychosocial stresses (job stress, social isolation), or health issues may all have an impact on both exposure to risk factors and AL. These unmeasured variables may have influenced the results and should be included in future research to have a more complete knowledge of the link between environmental and lifestyle exposures and AL. Also, the reliance on self-reported lifestyle habits, notably alcohol intake and smoking, increases the likelihood of errors in measurement. Participants may underreport or overreport their consumption habits due to social desirability bias or recollection difficulties. This could lead to misclassification, diluting the observed connections. Similarly, exposure to environmental toxins was assessed at a particular period, which may not accurately reflect long-term exposure levels. Future research should include biomarker measurements throughout time to obtain more accurate estimates of chronic exposure.

### 4.5. Future Direction

Future research should prioritize longitudinal studies to better understand how the cumulative effect of different exposures and lifestyle choices affect allostatic load (AL) over time thereby establishing temporal correlations and revealing the lagged effects of chronic stressors. These studies could also incorporate repeated biomarker measurements over time to provide a more accurate assessment of cumulative exposures. This approach would also allow for the investigation of exposure thresholds for lead, mercury, and smoking, which may have more substantial effects on AL when measured over longer periods of time or at greater levels. Understanding these thresholds can help improve risk assessments and direct targeted interventions. Furthermore, integrating advanced analytical techniques, such as speciation analysis for metals and building integrated models that utilize sophisticated approaches, such as machine models to capture non-linear interactions, may enhance predictions of how different combinations of stressors contribute to AL. Such models can account for non-linear interactions and synergistic effects among stressors, increasing the capacity to identify susceptible populations and maximize measures for lowering the cumulative burden of environmental and lifestyle risk factors.

## 5. Conclusions

This study revealed how lifestyle habits like alcohol consumption and smoking, along with environmental exposures to lead, cadmium, and mercury, affect allostatic load (AL), a measure of chronic stress. Among these factors, alcohol consumption was the most important contributor to increased AL. Alcohol had a strong and steady link with higher AL, showing how it disrupts the body’s ability to handle stress. Cadmium’s effects were more complex and non-linear, especially when combined with other exposures. On the other hand, lead, mercury, and smoking had smaller individual effects on AL. However, when combined with stronger stressors like alcohol, their impact may grow. This highlights the importance of examining how multiple exposures work together to influence stress. The findings from this study highlight the need for public health strategies that focus on reducing alcohol consumption, helping people quit smoking, and lowering exposure to harmful metals like cadmium. These efforts should especially target vulnerable groups, such as those with fewer resources, who often face multiple stressors at once. Future studies should look at longitudinal data to better understand how these factors cause stress and to identify the levels of exposure that pose the greatest risks.

## Figures and Tables

**Table 1:** Descriptive statistics for continuous variables.

Variable	N	Geometric Mean	Geometric Std. Dev.	Min	Max
Lead (µg/dL)	4379	1.05	1.82	0.05	42.5
Cadmium (µg/dL)	4379	0.370	1.97	0.07	13.0
Mercury (µg/L)	4379	1.42	1.47	0.20	63.6
AL (Allostatic Load)	1995	3.43	1.16	0	10.0
Age (Years)	4541	43.1	1.32	18	80.0

**Table 2:** Frequency distribution of categorical variables.

Variable	Category	Frequency	Percent
Sex	Male	2315	50.98
	Female	2226	49.02
Race/Ethnicity	Mexican American	626	13.79
	Other Hispanic	419	9.23
	Non-Hispanic White	1690	37.22
	Non-Hispanic Black	1065	23.45
	Non-Hispanic Asian	491	10.81
	Other Race/Multi-Racial	250	5.51

**Table 3:** Linear regression results for allostatic load (AL).

Variable	* Coefficient (β)	Std. Error	t-Value	*p*-Value	95% Confidence Interval
Alcohol	0.0933	0.0286	3.26	0.001	0.0369 to 0.1497
Smoking	0.0129	0.0194	0.66	0.508	−0.0086 to 0.0345
Lead	−0.1056	0.0744	−1.42	0.157	−0.2518 to 0.0408
Cadmium	−0.0001	0.1034	−0.00	0.999	−0.2037 to 0.2036
Mercury	−0.0149	0.0521	−0.29	0.773	−0.1175 to 0.0877

* Adjusted for age, gender, race/ethnicity, and income.

**Table 4:** BKMR analysis of allostatic load: group and conditional posterior inclusion probabilities for lead, cadmium, and mercury.

Variable	Group	Group PIP	Conditional PIP
lead	1	0.386	0.175
cadmium	1	0.386	0.753
mercury	1	0.386	0.072
alcohol	2	0.999	0.999
smoking	2	0.999	0.001

## Data Availability

The data presented in this study are openly available on the CDC NHANES site at https://wwwn.cdc.gov/nchs/nhanes/continuousnhanes/overview.aspx?BeginYear=2017 (accessed on 30 October 2024).
